# Lush Ice High: A Rare Case of Nicotine E-Liquid-Induced Toxic Encephalopathy

**DOI:** 10.7759/cureus.77486

**Published:** 2025-01-15

**Authors:** Shebin A George, Michelle Braha, Sahar N Chaudhary, Muhammad A Aziz

**Affiliations:** 1 Internal Medicine, Florida International University, Herbert Wertheim College of Medicine, Miami, USA

**Keywords:** biphasic syndrome, e-cigarettes, nicotine, public health, toxic encephalopathy

## Abstract

E-cigarette consumption has increased globally, partly due to its perceived role as a harm-reduction alternative to conventional cigarettes. It has been linked to various neurotoxic pathologies including stroke, cognitive dysfunction, and neurodevelopmental disorders. Acute e-liquid intoxication can lead to a fatal syndrome of respiratory failure, cardiovascular instability, acute encephalopathy, and gastrointestinal distress. We present a case of toxic encephalopathy due to e-liquid intoxication directly from an e-cigarette device. Our patient is a 59-year-old man who presented to the hospital with signs of tachycardia, tremors, paranoia, disorientation, hypervigilance, physical hostility, and aphasic mutism, was unable to provide any history, and was treated on suspicion of acute encephalopathy and possible delirium tremens. Imaging including CT brain and chest X-ray was unremarkable. Laboratory tests were significant for leukocytosis (16.4 x 10^3^/mcL), hypernatremia (157 mmol/L), acute kidney injury (blood urea nitrogen (BUN) 26 mg/dL, creatinine 1.70 mg/dL), anion gap metabolic acidosis (18 mEq/L), and urine toxicology screen positive for cocaine and benzodiazepines. He was thereafter managed in the intensive care unit for close monitoring with an uncomplicated course. On the second day, he was found to be awake, alert, oriented, and without any focal neurological deficits. The patient claimed that prior to the emergency room (ER) visit, he orally consumed 4 mL of e-liquid from a 5% nicotine e-cigarette pen through an opening in the device. Serum analysis was significant for cotinine concentration of 30 ng/mL (primary metabolite of nicotine) and nicotine concentration of 2 ng/mL. No symptomatic sequelae were reported for the rest of the hospital course, and the patient was discharged on the fourth day after laboratory tests showed a resolution of earlier findings. This case of moderate e-liquid intoxication showed acute encephalopathy with resolution significantly different from the classically described biphasic pattern of nicotine intoxication-an initial stimulatory syndrome of nausea, hypertension, tachycardia, tremor, and seizures followed by depressor symptoms including hypotension, bradycardia, weakness, and CNS and respiratory depression. The neurotoxic effects of nicotine and non-nicotinic substances in e-cigarettes need to be investigated further to develop standardized management guidelines for acute e-liquid intoxication.

## Introduction

The use of e-cigarette devices (or vape pens) has been increasing globally with an estimated prevalence rate of 18% among young adults [1]. Although its popularity can be attributed to its perceived role as a harm-reduction alternative to conventional cigarettes, the contents of e-cigarettes have long been implicated for their injurious effects on health [2]. While the long-term effects of these devices remain to be investigated, it has been linked to, among others, neurotoxic pathologies including stroke, cognitive dysfunction, and neurodevelopmental disorders [3].

The availability of nicotinic e-liquid refill containers provides a non-combustible concentrated form with the potential for abuse and intoxication. Previous case reports have described acute intoxication of e-liquids presenting with a syndrome of respiratory failure, cardiovascular instability, acute encephalopathy, and gastrointestinal distress, leading to a high rate of fatalities [4]. The severity of symptoms ranges from mild to severe and has been postulated to typically present in a biphasic manner-an initial prodrome of hypertension, tachycardia, headache, nausea, vomiting, abdominal pain, ataxia, tremor, and seizures, followed by sequelae of bradycardia, hypotension, CNS depression, respiratory failure, and cardiac arrest [5].

The vast majority of cases previously reported describe acute intoxication secondary to suicidal intention with consumption of e-liquid present in refill containers [4]. In our report, we present one of the few reported cases of e-liquid intoxication directly from an e-cigarette device with no suicidal intention. We also discuss the subsequent presentation of toxic encephalopathy, electrolyte disturbances, and resolution.

## Case presentation

A 59-year-old male patient was brought by emergency medical services to the emergency department with agitation and aggression, requiring physical restraints. At the presentation, he showed signs of tachycardia, tremors, paranoia, disorientation, hypervigilance, physical hostility, and aphasic mutism and was unable to provide any history. He had an extensive history of medication non-compliant schizoaffective disorder (bipolar type), alcohol abuse, and chronic crack cocaine abuse. He previously presented to an outside facility, where he exhibited tremulousness, disorientation, and diaphoresis and was administered midazolam, lorazepam, and droperidol upon suspicion of delirium tremens prior to transfer to our hospital. Thereafter, he ceased being agitated and was found mute and lethargic, yet responsive to verbal stimuli upon arrival. CT brain imaging and chest X-ray were unremarkable for prominent findings. EKG was significant for sinus tachycardia. Laboratory tests were significant for leukocytosis, hypernatremia, acute kidney injury, anion gap metabolic acidosis, and urine toxicology screen positive for cocaine and benzodiazepines. The patient was administered Lactated Ringers intravenous fluids and was then admitted to the intermediate medical care unit (IMCU) for close monitoring.

On the second day of admission, the patient was found to be calm, cooperative, and neurologically unremarkable during examination. He was awake, alert, and oriented to person, place, and time. Laboratory tests showed significant yet gradual resolution of the prior day’s findings. He vehemently denied any alcohol or cocaine use in recent days leading up to this episode. He reported that before the emergency room visit, he consumed approximately 4 mL of “Lush Ice” flavored e-liquid from a 5% nicotine vape pen (amounting to 200 mg of nicotine) because he was craving nicotine products and wanted to experience intoxication. He vehemently denied any suicidal intent behind his actions. He initially tried to inhale combustible smoke from the pen and, upon failure, resorted to drinking the e-liquid through an opening in the device. As per an eyewitness family member, he immediately experienced generalized motor weakness, dysarthria, and diaphoresis, which prompted them to call emergency medical services. The patient stated that he felt better than the previous day and denied any psychiatric symptoms although he was non-compliant with his home regimen of fluoxetine, doxepin, and naltrexone. Throughout this admission, the medical team did not ascertain any suicidal ideation or intention, or any psychiatric symptoms including delusions, hallucinations, or manic/depressive episodes. Since the medical team suspected e-liquid intoxication based on the patient's testimony, a blood serum sample was obtained on Day 2 and sent for analysis. However, since the hospital lacked the facilities to test for nicotine metabolites, the sample was sent to an outside facility that analyzed the sample nine days after the patient's initial admission. As reported, serum analysis was significant for a cotinine concentration of 30 ng/mL (primary metabolite of nicotine commonly used as a biomarker for tobacco smoke exposure) and a nicotine concentration of 2 ng/mL. The patient continued admission for two more days during which he had complete resolution of previous abnormal lab findings and symptoms and was then discharged after clearing psychiatry evaluation.

The critical laboratory values from all days of admission are presented in Table 1.

**Table 1:** Laboratory values during admission AST: aspartate transaminase; ALT: alanine transaminase

	Reference ranges	Day 1	Day 2	Day 3	Day 4
Blood gas					
pH	7.35-7.45	7.34	-	7.44	7.4
pCO_2_ (mmHg)	35.0-45.0	37	-	39	46
pO_2_ (mmHg)	75.0-100.0	52	-	38	108
HCO_3_ (mmol/L)	22.0-28.0	20	-	26	28
Hematology					
WBC (x10⁹ cells/L)	4.0-11.0	16.4	13	9.9	7.6
General chemistry					
Sodium (mEq/L)	135.0-145.0	157	146	140	141
Potassium (mEq/L)	3.5-5.0	4	4.3	3.9	4.3
Anion gap (mEq/L)	8.0-16.0	18	10	7	8
Blood urea nitrogen (mg/dL)	7.0-20.0	26	24	14	11
Creatinine (mg/dL)	0.7-1.3	1.7	0.8	0.6	0.6
Total protein (g/dL)	6.0-8.3	8.7	-	-	5.8
Albumin (g/dL)	3.5-5.0	5.2	-	-	3.3
AST (IU/L)	10.0-40.0	35	-	-	30
ALT (IU/L)	7.0-56.0	30	-	-	22
Alkaline phosphatase (IU/L)	44.0-147.0	80	-	-	55
Magnesium (mg/dL)	1.7-2.2	3	2.4	1.9	1.7
Ammonia (µmol/L)	15.0-45.0	19	-	-	-
Creatine phosphokinase (IU/L)	38.0-174.0	250	-	320	-
Lactic acid (mmol/L)	0.5-2.2	1.4	-	-	-
Glucose (mg/dL)	70.0-99.0	150	140	109	93

## Discussion

We believe our patient experienced significant yet relatively moderate symptoms compared to previously reported e-liquid intoxication cases due to the source of e-liquid, limited container volume, and route of intake. Although the initial drug screen was positive for cocaine and benzodiazepines, we do not suspect their role contributed to our patient's presentation. The administration of midazolam and lorazepam on Day 1 for suspected delirium tremens is more likely to be responsible for the positive benzodiazepine result and cannot explain the current presentation, particularly given the lack of a history of benzodiazepine use. Although our patient has a history of chronic cocaine abuse, the patient's presentation at admission is poorly explained by acute cocaine toxicity. More importantly, we can establish a strong temporal association due to the eyewitness testimony that reported the symptoms' onset immediately after the patient consumed the e-liquid. Our patient also experienced large-volume dehydration, likely due to the high salt content in the e-liquid or the nicotine itself, which resulted in moderate hypernatremia and acute kidney injury [6,7]. The elevated anion gap metabolic acidosis can be attributed to the effects of non-nicotinic solvents including propylene glycol and glycerin present in the e-liquid [8]. These findings were immediately resolved with the administration of Lactated Ringer fluids.

A previous case reported that a patient who intravenously injected 10 mL of e-liquid experienced lactic acidosis with elevated anion gap (likely secondary to propylene glycol) and coma, requiring mechanical ventilation [9]. Signs of nicotinic syndrome including tetraparesis, gaze palsy, and myoclonus were also noted. While our patient did exhibit moderate neurological signs including altered mental status, agitation, and aphasia, other commonly reported signs including ophthalmoplegia, neuromuscular dysfunction, and seizures were not present. We suppose the severity of symptom presentation is largely driven by the amount and dose of ingested e-liquid as well as the route of consumption. Another case described that a patient who orally ingested e-liquid subsequently developed severe hypoxic respiratory failure secondary to aspirating liquid as well as nicotinic toxicity [10]. Although our patient presented with signs of agitation and mild hypoxia, no signs of respiratory distress were noted, and the chest imaging was not significant for pulmonary insults. We foresee the risk of aspiration to be relatively high in such cases, which may complicate inpatient management.

Our patient reported that he orally consumed 4 mL of 5% nicotine e-liquid, estimated to be 200 mg of nicotine. While the delay in serum analysis for nicotine metabolites is a limitation, we can strongly infer that the serum levels align with the amount of e-liquid the patient reported consuming based on the known nicotine and cotinine metabolism rates [11]. Previous literature reported that survivors of acute intoxication had a first measured plasma nicotine concentration of 307 ± 312 µg/L whereas non-survivors had a nicotine concentration of 3,360 ± 1,692 µg/L (p < 0.05) [3]. On the other hand, cotinine was not found to be a reliable predictor of lethal effects in intoxicated patients. Survivors of acute intoxication had a first measured plasma cotinine concentration of 1,224 ± 971 µg/L whereas non-survivors had a concentration of 1,044 ± 731 µg/L (n.s.). It can be reasonably assumed that e-liquid consumption directly from e-cigarette devices does not translate to lethal concentrations of nicotine, attributed to its prepackaged size and e-liquid nicotinic concentration. This case also disputes the widely considered lethal nicotine dose of 60 mg [12]. However, even mild concentrations can produce serious encephalopathic and metabolic symptoms, as clearly seen in our patient.

Currently, there is no strong consensus on the lethal dosage of e-liquids, and there is a varied manifestation of symptoms previously reported. However, symptom severity and eventual cardiopulmonary collapse have generally been attributed to the extent of nicotinic acetylcholine receptor overstimulation [13]. The acute encephalopathic presentation of disorientation, tremulousness, and mutism was particularly prominent in our patient’s presentation. A commonly described biphasic toxidrome with parasympathetic symptoms including hypotension, CNS depression, and cardiac arrest was not seen in our patient who only experienced sympathetic symptoms [5]. We believe that this may suggest that a higher dose of nicotine may be required to activate the parasympathetic nicotinic receptors. While nicotinic stimulation has been shown to trigger symptoms, other e-liquid ingredients including propylene glycol, vegetable glycerin, free radicals, flavor additives, and heavy metals have also been implicated to cause neurotoxicity [3]. These compounds have been linked to calcium dyshomeostasis, epigenetic changes, impaired autophagy, impaired neurotransmission, mitochondrial dysfunction, neuroinflammation, and oxidative stress. In a previous study, the extent of cytotoxicity was found to be dependent on non-nicotinic toxicants in e-liquids rather than the concentration of nicotine, likely via the penetration of the blood-brain barrier and subsequent neural remodeling [14,15]. Exposure to non-nicotinic toxicants was also linked to impaired neuromotor activity, cognition, learning, and memory in animal studies [16]. Direct intoxication of e-liquid increases the risk of non-nicotinic intoxication as it bypasses the e-cigarette apparatus, which regulates the concentration of these compounds [17]. The role of non-nicotinic e-liquid toxicants in producing neurological sequelae seen in our patient is largely unknown, and it remains an interesting area of future investigation.

This patient interaction in the emergency department highlighted how the possibility of e-liquid-induced toxicity was not considered in the presentation of altered mental status with a background of psychiatric disorder and substance abuse. This may have potentially resulted in further patient deterioration and morbidity. We recommend standardized inclusion and quantitative measurement of nicotine and its metabolites within the urine toxicology screening panel. Quantitative nicotinic levels may serve as useful indicators for impending biphasic toxidrome and/or morbidity. As the popularity of e-cigarettes increases, healthcare providers need to be prompt and thorough in their evaluation and management of e-liquid intoxication.

## Conclusions

Our patient presented with a rare case of e-liquid intoxication directly from an e-pen resulting in a syndrome of neurological and electrolyte disturbances with eventual resolution. While the harmful effects of e-cigarettes and their contents have been studied, there is still a paucity of literature, especially as it presents in acute intoxication, and the various forms of e-liquid consumption. As the use of e-cigarettes continues to rise, calls for stricter oversight and robust management guidelines to prevent fatalities are warranted.

## References

[1] Erhabor J, Boakye E, Obisesan O (2023). E-cigarette use among US adults in the 2021 Behavioral Risk Factor Surveillance System survey. JAMA Netw Open.

[2] Allbright K, Villandre J, Crotty Alexander LE (2024). The paradox of the safer cigarette: understanding the pulmonary effects of electronic cigarettes. Eur Respir J.

[3] Ruszkiewicz JA, Zhang Z, Gonçalves FM, Tizabi Y, Zelikoff JT, Aschner M (2020). Neurotoxicity of e-cigarettes. Food Chem Toxicol.

[4] Maessen GC, Wijnhoven AM, Neijzen RL (2020). Nicotine intoxication by e-cigarette liquids: a study of case reports and pathophysiology. Clin Toxicol (Phila).

[5] Scarpino M, Rosso T, Lanzo G (2021). Severe neurological nicotine intoxication by e-cigarette liquids: systematic literature review. Acta Neurol Scand.

[6] Esteban-Lopez M, Perry MD, Garbinski LD (2022). Health effects and known pathology associated with the use of E-cigarettes. Toxicol Rep.

[7] Herman M, Tarran R (2020). E-cigarettes, nicotine, the lung and the brain: multi-level cascading pathophysiology. J Physiol.

[8] Zar T, Graeber C, Perazella MA (2007). Recognition, treatment, and prevention of propylene glycol toxicity. Semin Dial.

[9] Belkoniene M, Socquet J, Njemba-Freiburghaus D, Pellaton C (2019). Near fatal intoxication by nicotine and propylene glycol injection: a case report of an e-liquid poisoning. BMC Pharmacol Toxicol.

[10] Jude J, Hiller H, Miller J (2021). Melon with a twist: a case of nicotine overdose after ingestion and aspiration of vape liquid. Mil Med.

[11] Benowitz NL, Hukkanen J, Jacob P 3rd (2009). Nicotine chemistry, metabolism, kinetics and biomarkers. Handb Exp Pharmacol.

[12] Mayer B (2014). How much nicotine kills a human? Tracing back the generally accepted lethal dose to dubious self-experiments in the nineteenth century. Arch Toxicol.

[13] Henstra C, Dekkers BG, Olgers TJ, Ter Maaten JC, Touw DJ (2022). Managing intoxications with nicotine-containing e-liquids. Expert Opin Drug Metab Toxicol.

[14] Bahl V, Lin S, Xu N, Davis B, Wang YH, Talbot P (2012). Comparison of electronic cigarette refill fluid cytotoxicity using embryonic and adult models. Reprod Toxicol.

[15] Heldt NA, Seliga A, Winfield M (2020). Electronic cigarette exposure disrupts blood-brain barrier integrity and promotes neuroinflammation. Brain Behav Immun.

[16] López-Ojeda W, Hurley RA (2024). Vaping and the brain: effects of electronic cigarettes and e-liquid substances. J Neuropsychiatry Clin Neurosci.

[17] Lee YJ, Na CJ, Botao L, Kim KH, Son YS (2020). Quantitative insights into major constituents contained in or released by electronic cigarettes: propylene glycol, vegetable glycerin, and nicotine. Sci Total Environ.

